# Pain Assessment and Documentation for Older Adults Presenting with Non-surgical Conditions in Emergency Room

**DOI:** 10.1093/geroni/igab046.2284

**Published:** 2021-12-17

**Authors:** Raza Haque, Mara Bezerko, Lauren Tibbits, Karen Tate

**Affiliations:** 1 Michigan State University, East Lansing, Michigan, United States; 2 Michigan State University, College of Human Medicine, Michigan, United States

## Abstract

Pain is one of the most common reasons for Emergency Department (ED) visits among older adults. However, timely pain assessment and management in this population in ED is a challenging task due to many factors ranging from; sensory, cognitive impairments, chronic pain, reliability of assessment tools, multimorbidity and system factors such as triage-based dynamic ED workflow. Where the implementation of the EMR was anticipated to improve patientcare, literature has indicated the barriers in effective utilization of the EMR for this purpose. We posit that pain assessment and documentation could be variable among older adults presenting with non-surgical conditions. Objectives:1. To examine the proportion of documented initial pain assessment of nonsurgical older adults visiting emergency department 2. To examine the number of initial pain assessments documented in the chart by the five major categories of ICD-10 diagnoses upon discharge. Methods: A retrospective exploratory chart review of 4613 emergency room visits for first pain assessment in the EMR conducted for all adults 65 years or older, presenting with non-surgical conditions, who were discharged same day at an urban teaching hospital. Results: In our study 75.72% of encounters reviewed had a documented pain assessment. Completed pain assessments for the corresponding five most common non-surgical diagnostic categories presenting to our ED: Abdominal pain (92.59%), MSK (92.11 %), chest pain (83.92%), dyspnea (80%) and falls (79.46%). Conclusion: Frequency of pain assessment and the management process of older adults presenting with non-surgical conditions in the institution studied was variable and differed based on presenting conditions.

